# Convolutional Neural Network Based on Crossbar Arrays of (Co-Fe-B)*_x_*(LiNbO_3_)_100−*x*_ Nanocomposite Memristors

**DOI:** 10.3390/nano12193455

**Published:** 2022-10-03

**Authors:** Anna N. Matsukatova, Aleksandr I. Iliasov, Kristina E. Nikiruy, Elena V. Kukueva, Aleksandr L. Vasiliev, Boris V. Goncharov, Aleksandr V. Sitnikov, Maxim L. Zanaveskin, Aleksandr S. Bugaev, Vyacheslav A. Demin, Vladimir V. Rylkov, Andrey V. Emelyanov

**Affiliations:** 1 National Research Center “Kurchatov Institute”, 123182 Moscow, Russia; 2 Faculty of Physics, Lomonosov Moscow State University, 119991 Moscow, Russia; 3 Department of Solid State Physics, Faculty of Radio Engineering and Electronics, Voronezh State Technical University, 394026 Voronezh, Russia; 4 Moscow Institute of Physics and Technology, State University, 141700 Dolgoprudny, Russia; 5 Kotelnikov Institute of Radio Engineering and Electronics RAS, 141190 Fryazino, Russia

**Keywords:** memristor, resistive switching, nanocomposite, neuromorphic computing, convolutional neural network

## Abstract

Convolutional neural networks (CNNs) have been widely used in image recognition and processing tasks. Memristor-based CNNs accumulate the advantages of emerging memristive devices, such as nanometer critical dimensions, low power consumption, and functional similarity to biological synapses. Most studies on memristor-based CNNs use either software models of memristors for simulation analysis or full hardware CNN realization. Here, we propose a hybrid CNN, consisting of a hardware fixed pre-trained and explainable feature extractor and a trainable software classifier. The hardware part was realized on passive crossbar arrays of memristors based on nanocomposite (Co-Fe-B)_x_(LiNbO_3_)_100−x_ structures. The constructed 2-kernel CNN was able to classify the binarized Fashion-MNIST dataset with ~ 84% accuracy. The performance of the hybrid CNN is comparable to the other reported memristor-based systems, while the number of trainable parameters for the hybrid CNN is substantially lower. Moreover, the hybrid CNN is robust to the variations in the memristive characteristics: dispersion of 20% leads to only a 3% accuracy decrease. The obtained results pave the way for the efficient and reliable realization of neural networks based on partially unreliable analog elements.

## 1. Introduction

Memristor-based neuromorphic computing systems (NCSs) provide a fast, high-computational, and energy-efficient approach to neural network (NN) training and solving cognitive problems (pattern and speech recognition, big data processing, prediction, and so on) [1,2]. Memristors could be organized in large crossbar arrays (with critical dimensions down to 6 nm [3]) to perform vector–matrix multiplication in a natural one-step method by weighted electrical current summation (according to the Ohm’s and Kirchhoff’s laws) [4]. In contrast, being the most massively parallel operation in NN learning and inference, vector–matrix multiplication is extremely time- and energy-expensive in traditional von Neumann architectures [2]. Owing to this difference, memristor-based NCSs are of high interest. Memristors have already been successfully implemented for diverse NCS realizations, and such schemes as perceptrons [5,6], spiking [7,8], or long short-term memory [9] networks and others (including NN circuits of Pavlov’s associative memory) [10,11,12] have been demonstrated. Most of these NCSs are usually trained by various types of gradient descent learning algorithm, the hardware realization of which is challenging because of unreliable cycle-to-cycle (c2c) and device-to-device (d2d) variations of memristive devices [2]. Several approaches have been proposed to partially mitigate these problems, including reservoir computing [13] and convolutional [14,15] NNs. The latter one is of particular interest as it allows to reduce the number of required weights (i.e., memristors) compared with fully connected NNs and, at the same time, demonstrates excellent performance in object recognition and image processing [15,16]. Convolutional NNs (CNNs) consist of two main parts: the feature extractor (convolutional layers) and classifier (fully connected layers). Convolutional layers extract feature maps from the input images by applying filters (kernels) of different dimensions, which allows decreasing the number of inputs. Most studies on memristor-based CNNs use either software models of memristors to emulate both parts of CNNs [16,17,18,19,20,21] or fully hardware parts of CNNs [14,22,23]. However, it significantly complicates the evaluation of the memristor-based convolutional layer efficiency, which should not be neglected. Generally, CNNs are prone to learning untrustworthy features and overfitting. An illustrative example of this case includes a CNN trained to classify images of huskies and wolves, which instead learned background features of the images, such as the presence of snow [24]. This highlights the importance of so-called explainability of the NNs. One possible way to control the feature extractor weights is to train the convolutional layers ex situ via a traditional computing system, and then transfer them to the memristors (hybrid training) [22]. Multiple software algorithms may help visualize and study the extracted features in this case [25]. However, this approach implies that a high-cost training process alongside with its verification should be performed in the software before the transfer of the convolutional kernel weights to the hardware. Moreover, the convolutional kernels may need to be retrained if the classification problem is changed.

In this work, we propose a general approach to the implementation of a memristor-based CNN—a hybrid network, consisting of a hardware fixed pre-trained and explainable feature extractor and a trainable software classifier. Unlike the hybrid training process, where the weight update of a hardware memristor-based NN is made according to the software ex situ training results at each training cycle [22], in our case, no additional training of the CNN memristive part is needed. The hybrid CNN possesses the advantages of both hardware systems in terms of energy and computational efficiency and software systems in terms of architectural flexibility. The usage of convolutional layers with universally recognized fixed kernels (horizontal and vertical) gives hope that such a hybrid network would be eligible for any given image classification problem, and only the weights of its classifier part would require some fine-tuning. Here, we test this approach on the Fashion-MNIST (F-MNIST) image recognition problem [26]. The main goal of this work is to estimate the efficiency of the fixed memristor-based convolutional layers compared with the ideal software trainable ones.

## 2. Materials and Methods

### 2.1. Device Fabrication 

**Figure 1:**
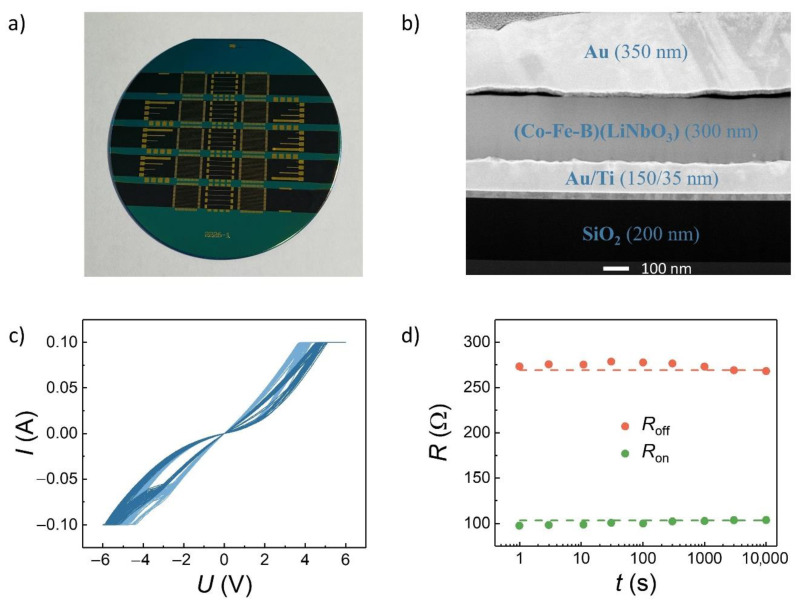
(**a**) Photo of a single silicon wafer with multiple 4 × 4 and 16 × 16 memristor crossbar arrays; (**b**) dark field TEM image of a single memristor of an array with denoted layers; (**c**) *I*–*V* characteristics of all utilized NC memristors with optimal *x* ≈ 23 at.% (*x* is the metal concentration in the LNO NC); (**d**) retention time of a single memristor from the crossbar array (only a few selected data points are presented for better visibility, dashed lines represent the average values of the resistances).

Arrays of crossbar M/NC/LNO/M memristors were created in a technological route based on laser photolithography on Heidelberg 66fs lithograph (patterning electrode buses), ion-beam sputtering on the original system: in the beginning of target LiNbO_3_ (≈10 nm), and then composite target (Co_40_Fe_40_B_20_)*_x_*(LiNbO_3_)_100__−*x*_ with *x* ≈ 10–25 at.% (≈230 nm). Plasma chemical deposition via Trion Oracle III was used for deposition of the isolating Si_3_N_4_ layer to isolate the edge regions of memristors in the array from possible electrical breakdown effects (see details in [27]). Multiple crossbar arrays of different sizes were created on one silicon wafer in one technological cycle (Figure 1a), and one of them (16 × 16) was used in this work.

### 2.2. Electrical Measurements 

Electrophysical characterization of memristors as well as their switching to different required resistance states during the preparation of convolutional kernels were performed using the source measurement unit (National Instruments PXIe-4140). To provide electrical signals, representing pixels of image, a digital-to-analog converter (National Instruments PXIe-6738) was used. Output currents (the results of convolutional operations) were measured using a two-channel digital oscilloscope (National Instruments PXIe-5110). For *I*–*V* measurements, the compliance current was set to 100 mA for both voltage polarities and the rate of voltage scan was set to 2 V/s.

### 2.3. TEM

The structural investigations were carried out in a scanning/transmission electron microscope (S/TEM) Osiris (Thermo Fisher Scientific, Carlsbad, CA, USA), equipped with a high angle annular dark field (HAADF) detector (Fischione, Pittsburgh, PA, USA) and an energy-dispersive X-ray (EDX) Bruker Super-X spectrometer (Bruker, Billerica, MA, USA) for the chemical composition analysis.

### 2.4. Hardware Convolutional Layer Implementation

The kernels were implemented on a crossbar array as follows. As long as memristors cannot provide negative weights directly, the kernels were split into two parts—with positive and negative weights only. They defined target resistances of memristors in two columns: devices, corresponding to non-zero weight (1 for the first column and −1 for the second one) were set to *R*_on_ state, while others (−1 and 0 for the first column and 0 and 1 for the second one) were set to *R*_off_ state. Each memristor was set to the target state individually, performing the corresponding half of the *I*–*V* loop (positive polarity for the *R*_on_ state, and vice versa). Vertical electrodes were grounded via two equal resistors; this way, the current-to-voltage conversion was performed to measure output currents using an oscilloscope. When each patch of the image is fed to the crossbar array, these output currents from two columns represent the result of convolutional operation owing to intrinsic properties of the crossbar array geometry—input voltages, i.e., the pixels of the patch, are weighted with memristor conductance, i.e., the weights of the convolutional kernel. As stated in the main text, the resulting output current equaled to the difference between the currents from two array columns. In order to speed up the work, the resulting currents for every possible 3 × 3 binarized image patch (total of $2^{3\times 3}=512$ possible patches) were measured. After all 512 currents had been measured, they were normalized to a [0,1] interval and used in the software classifier. These operations were performed for horizontal and vertical filters successively.

### 2.5. Neural Network Simulation

Initial data preprocessing included images’ binarization, i.e., each pixel of the image with a higher value than some threshold value equaled 0, while all others equaled 1. The threshold value equaled 10 and was estimated by eye, in order not to make it another hyperparameter of the CNN. The F-MNIST dataset is pre-divided into training (60,000 images) and testing (10,000 images) datasets. The original training dataset was then divided into training (50,000) and validation (10,000) datasets. The CNN hyperparameters were adjusted with the validation dataset, while the test dataset was used only for the final evaluation of the CNN performance. The hyperparameters list and detailed discussion of their fitting are presented in Supplementary Note S1 (Figures S1–S3, Table S1). The CNN was simulated in the PyTorch framework.

## 3. Results and Discussion

In this work, the LiNbO_3_ (LNO)-based memristors were used, as they are of emerging interest [28,29,30], especially those with embedded metal nanogranules [31]. In our studies, the capacitor metal/nanocomposite/metal (M/NC/M) structures based on (Co-Fe-B*)_x_(*LiNbO_3_*)*_100__−*x*_ NC were fabricated by ion-beam sputtering with a metal content *x* ≈ 8–25 at.% [31]. The NC films, along with the metal nanogranules of 3–6 nm, contained a large number of dispersed Co (Fe) atoms (up to ~10^21^–10^22^ cm^−3^). At some optimal *x* ≈ 8–15 at.%, the M/NC/M structures manifest stable resistive switching (RS) through a multifilamentary mechanism [32]; demonstrate high endurance, long retention, and multilevel RS [33,34]; and can be successfully used in NCSs [27,35].

In this work, we fabricated NC structures with a thin built-in LNO layer near the bottom electrode (i.e., structures like M/NC/LNO/M), which plays a critical role in the realization of stable RS [32]. The arrays of the NC memristors were fabricated in a 16 × 16 crossbar architecture (Figure 1a). Then, their characteristics were studied in order to verify the eligibility of the NC crossbar memristors for the hardware realization of different 3 × 3 convolutional kernels.

The results of transition electron microscopy (TEM) provided a descriptive picture of the layer thickness (Figure 1b) and composition (elemental maps are presented in Figure S4) for a single memristive element from a 16 × 16 crossbar array. High-resolution images (Figure S5) confirmed the presence of a ~10 nm thick pure LNO layer near the bottom electrode. Figure 1c presents the current–voltage (*I*–*V*) characteristics of all 18 memristors (9 rows and 2 columns) used in this work, five cycles for each one. As can be seen in Figure 1c, while c2c variations for these memristors are negligible, the d2d variations are more pronounced. Two groups of devices could be selected from the *I*–*V* curves (shaded in different colors in Figure 1c). Each group represents devices from different columns of the crossbar array. The variations in the resistive switching voltage of these groups are associated with different resistances of crossbar busses, which act as load resistances, and some additional voltage drops on them. The bus resistances should be reduced to decrease the d2d variations in a crossbar array. However, as shown below, all of these devices can operate as equivalent parts of a convolutional kernel. Another important memristive characteristic for the convolutional layer implementation is retention time—after the weights of all memristors are adjusted to represent the chosen kernel, they should not vary. As can be seen from Figure 1d, the resistance drift from both initial states (high resistance state, *R*_off_, and low resistance state, *R*_on_) is negligible compared with their difference *R*_off_—*R*_on_. It should be noted that the resistance values of the obtained memristors are not high enough (≤1 kΩ), probably because of the small thickness of the LNO layer in the M/NC/LNO/M structures under study.

**Figure 2:**
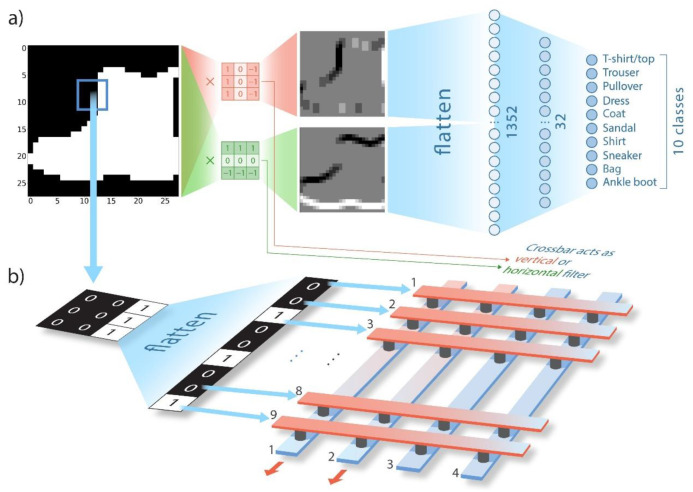
Schematic illustration of (**a**) the proposed neural network architecture and (**b**) the hardware convolutional layer implementation on the memristive crossbar array.

Figure 2a illustrates the proposed NN architecture. The original F-MNIST images were binarized in order to simplify the implementation of the hardware part. The features were then extracted with either a horizontal/vertical filter or both at once (the example of the extracted features is presented in Figure 2a). Then, the obtained feature matrices were flattened, normalized, and fed to the fully connected classifying layers (676 input neurons in the case of the CNN with one filter and 1352 in the case of the CNN with two filters). Figure 2b demonstrates the hardware feature extractor implementation. The image was divided into 3 × 3 patches; each patch was then flattened and fed to the crossbar array (i.e., the corresponding voltages were applied, 1 V for “1” pixels of the patch and 0 V for “0” pixels). The crossbar array acted either as a horizontal or a vertical kernel, i.e., the weights of nine memristive devices were adjusted to represent the chosen normalized and flattened 3 × 3 kernel. In order to obtain negative weights of the kernels, two columns of the crossbar array were used (i.e., nine rows-inputs and two columns-outputs were used in this work, as specified in the figure), and the resulting output current equaled the difference between the currents from both columns. A more detailed discussion of the feature extractor implementation can be found in Section 2.

**Figure 3:**
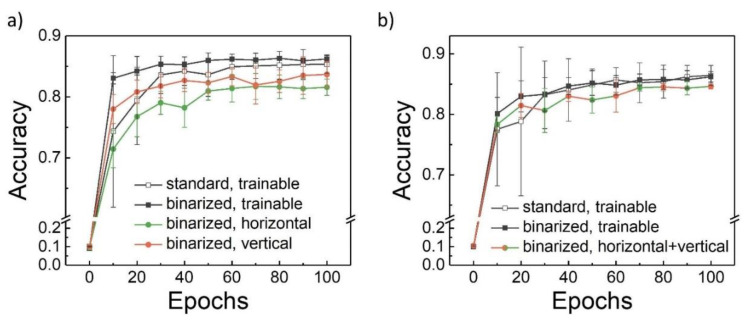
Software modeling of the convolutional neural network with (**a**) one and (**b**) two kernels (filters). The image parameters (standard or binarized image) as well as the kernel weight parameters (trainable or fixed weights) are specified in the graphs. Each curve was obtained 10 times; the mean values with their standard deviations are presented in the graphs.

The proposed NN architecture (Figure 2a) was additionally simulated in the software for subsequent comparison with the hybrid NN results. Figure 3a presents the accuracy of the one-filter NN during training, estimated on a validation dataset (the discussion of the initial dataset portioning can be found in Section 2). Figure 3b demonstrates the results for the two-filter NN. Three main conclusions can be drawn from these figures. Firstly, the binarization of the images does not lead to an accuracy decrease, so such a simplification can be done. Secondly, the CNNs with fixed filters do not concede to the trainable ones dramatically. Finally, during the first training epochs, the trainable filter usage leads to high accuracy variations; thus, such CNNs concede to the CNNs with fixed filters at the beginning of the training process. These results authorized the creation of a hybrid CNN.

**Figure 4:**
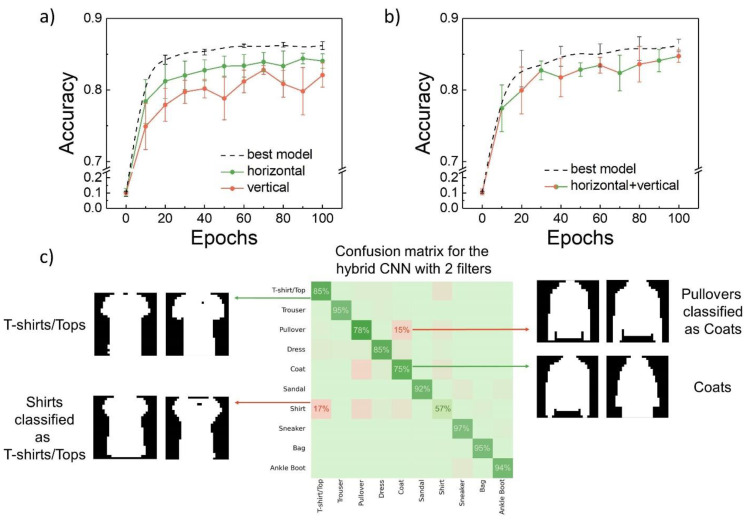
Comparison of the smoothed software modeling results to the hybrid NN ones with (**a**) one and (**b**) two kernels (filters). (**c**) Confusion matrix for the hybrid CNN with two filters, evaluated on the test dataset, with examples of the misclassified images for the classes with the most frequent mistakes. The maximum ideal values for the main diagonal items equal 100%.

Figure 4a,b compare the results obtained from the hybrid NNs and full software systems with one and two filters correspondingly. The best simulation results from Figure 3a,b were chosen to make the comparison under stringent conditions. It can be seen that the hybrid NNs with one filter considerably concede to the simulated NN with a trainable filter. However, the results after 100 epochs for the two-filter hybrid NN are comparable to the simulation results. A more complete set of features, obtained using both horizontal and vertical filters, was generated for each image in this case, leading to a higher accuracy score. This result raises hope that some expanded set of filters may be created, which would extract all of the most important features from the images without additional training of the filters, thus leading to the creation of a generic hybrid NN with enhanced classification accuracy.

Generally, the F-MNIST dataset was developed in order to replace the conventional MNIST digit dataset. Some modern software NNs can classify the MNIST digit dataset with an accuracy >99%, which makes it too simple for the NN performance evaluation [36]. In contrast, quite elaborate software NN architectures are required in order to surpass 90% accuracy for the F-MNIST dataset [26,37]. Meanwhile, the number of trainable parameters for such NNs equals 500–700 k (grayscale F-MNIST). The same goes for the memristor-based CNNs, e.g., sixteen 9 × 9 convolutional kernels were needed to reach ~87% accuracy (binarized F-MNIST) [16]. Another memristive CNN example demonstrated ~93% with ~3.5 M parameters (grayscale F-MNIST) [38]. In our study, the test classification accuracy of the hybrid CNN with two filters equaled ~ 84%, while the number of trainable parameters equaled ~44 k. This is a high enough accuracy value for a hybrid system with such a small number of parameters. Most mistakes were made for the classes, which are almost indistinguishable in the case of the binarized images (Figure 4c). The reduced number of trainable parameters leads to a less sophisticated and more robust training process, so introduction of the memristors does not dramatically decrease the classification accuracy.

Moreover, the influence of the variability introduction to the hardware CNN part on its performance was studied. The weights of the hardware filters (i.e., resistive states of the memristive crossbar) in the hybrid CNN are set once and are not tuned in the future. Thus, memristors should remain in their initial resistive states to produce a reliable output from the feature extractor. It can be seen in Figure 1d that our memristors have good retention, i.e., their states are not changed in time even if no external voltage is applied. However, it is known that memristive systems are in general prone to variations in characteristics [2]. It is probable that, after a longer time, some variations may appear as a result of internal microscopic degradation or external impacts. Therefore, we examine the influence of such possible variations on the classification accuracy. In order to simulate the variation introduction to the CNN, each output current from the memristive convolutional layer was chosen from a normal distribution, for which the mean value equaled the experimental results and the coefficient of variation was chosen from 0 to 100%. Only a high coefficient of variation (e.g., 100%) led to a considerable degradation of the training process and a decrease in the test dataset accuracy. All obtained data are summarized in Table S2 and demonstrate the robustness of the hybrid CNN to the variations in the memristor characteristics: dispersion of 20% leads to only a 3% decrease in accuracy.

## 4. Conclusions

In summary, we fabricated and studied the crossbar arrays of nanocomposite-based (Co-Fe-B)_x_(LiNbO_3_)_100−x_ memristors. Memristors in a single crossbar array demonstrate negligible c2c variations, while d2d variations are more pronounced, which was attributed to the considerable impact of the crossbar busses’ resistances to the total resistance of the structure. Using the nanocomposite-based crossbar arrays, we implemented a hybrid CNN, consisting of a hardware feature extractor with one/two kernels and a software classifier. The two-kernel CNN was able to classify the binarized Fashion-MNIST dataset with an accuracy of ~84%. The performance of the hybrid CNN is comparable to the full software and full hardware (memristive) systems, while the number of trainable parameters for the hybrid CNN is substantially lower. Moreover, the hybrid CNN is shown to be robust to the variations in memristive characteristics. The obtained results raise hope that enhanced performance may be achieved for any given image classification task in the future, if some expended set of fixed kernels is created for the hybrid CNN.

## Data Availability

The utilized dataset and Pytorch framework are publicly available. The CNN architecture and its hyperparameters are provided in the text, which simplifies the replication of the software modeling results. The other data that support the findings of this study are available from the corresponding author upon reasonable request.

## Supplementary Materials

The following supporting information can be downloaded at: https://www.mdpi.com/article/10.3390/nano12193455/s1, Figure S1: All tested via Optuna hyperparameter values with the resulting objective values (accuracy scores). The best values of each hyperparameter are denoted with bolder and brighter lines; Figure S2: The relative importance of each hyperparameter of the CNN, calculated via Optuna; Figure S3: Software modeling of the CNN with binarized F-MNIST and trainable filter for different CNN hyperparameters (Optuna and manually fine-tuned); Table S1: The Optuna-optimized hyperparameters and manually fine-tuned hyperparameters; Figure S4: Elemental EDX-maps of the M/NC/LNO/M memristor from a crossbar array; Figure S5: High resolution dark field TEM image of the area near the bottom electrode (at the edge of the crossbar busses intersection) of the M/NC/LNO/M memristor from a crossbar array; Table S2. The influence of the introduced variation on the training process of the 2-kernel hybrid CNN. Reference [39] is cited in the Supplementary Materials.
