# Supplementary material for: Convolutional Neural Network Based on Crossbar Arrays of (Co-Fe-B)x(LiNbO3)100−x Nanocomposite Memristors
Source: Nanomaterials (Basel). 2022 Oct 3;12(19):3455. doi: 10.3390/nano12193455 (PMC9565409; doi:10.3390/nano12193455)
Supplement: Supplementary file 1 [file nanomaterials-12-03455-s001.zip › nanomaterials-1912956-supplementary/nanomaterials-1912956-supplementary.pdf]

Convolutional Neural Network Based on Crossbar Arrays of (Co-Fe-B)$_x$(LiNbO$_3$)$_{100-x}$ Nanocomposite Memristors

Anna N. Matsukatova [1,2], Aleksandr I. Iliasov [1,2], Kristina E. Nikiruy [1,†], Elena V. Kukueva [1], Aleksandr L. Vasiliev [1], Boris V. Goncharov [1], Aleksandr V. Sitnikov [1,3], Maxim L. Zanaveskin [1], Aleksandr S. Bugaev [4], Vyacheslav A. Demin [1], Vladimir V. Rylkov [1,5] and Andrey V. Emelyanov [1,4,*]

[1] National Research Center "Kurchatov Institute", 123182 Moscow, Russia
[2] Faculty of Physics, Lomonosov Moscow State University, 119991 Moscow, Russia
[3] Department of Solid State Physics, Faculty of Radio Engineering and Electronics, Voronezh State Technical University, 394026 Voronezh, Russia
[4] Moscow Institute of Physics and Technology, State University, 141700 Dolgoprudny, Russia
[5] Kotelnikov Institute of Radio Engineering and Electronics RAS, 141190 Fryazino, Russia
* Correspondence: emelyanov_av@nrcki.ru
† Present address: Technische Universtität Ilmenau, Ehrenbergstrasse 29, 98693 Ilmenau, Germany

Supplementary note 1

The hyperparameters were optimized via an automatic hyperparameter optimization framework Optuna based on the classification accuracy score for the validation dataset.[1] Fig.S3 depicts all tested hyperparameter values with the resulting objective values (accuracy scores). Fig. S4 depicts the relative importance of each hyperparameter in the CNN performance optimization. As long as learning rate appeared to be the most important hyperparameter, it was manually fine-tuned to further improve the CNN. The change of the second most important feature, neuron quantity, was not necessary for the CNN improvement, however was made in order to reduce the CNN architecture dimensions. This fine-tuning lead to a perceptible accuracy increase (Fig. S5). The values of Optuna-optimized hyperparameters along with the manually fine-tuned hyperparameters are presented in Table S1.

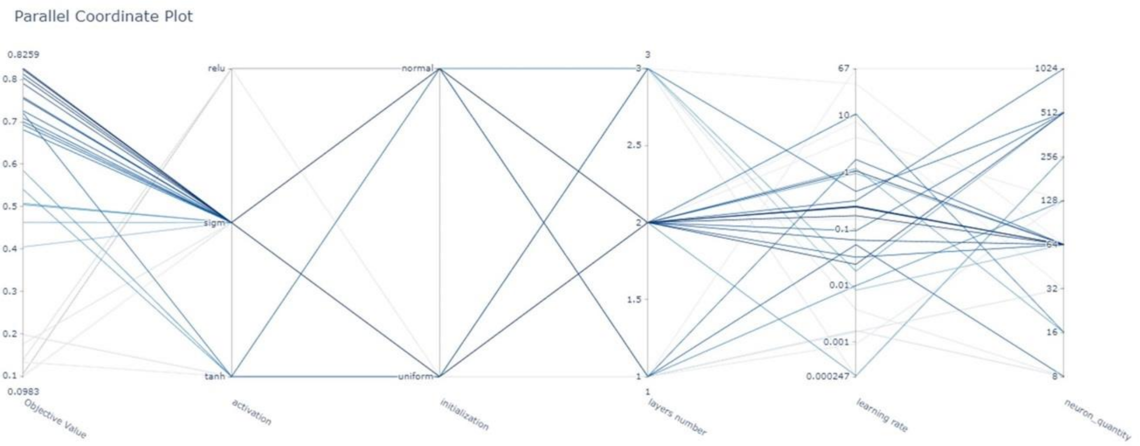

Figure S1. All tested via Optuna hyperparameter values with the resulting objective values

(accuracy scores). The best values of each hyperparameter are denoted with bolder and brighter lines.

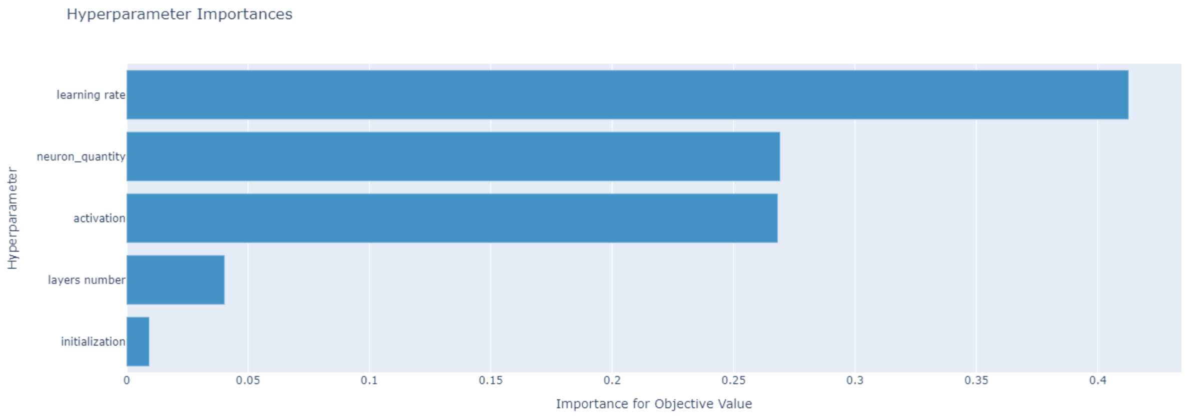

Figure S2. The relative importance of each hyperparameter of the CNN, calculated via Optuna.

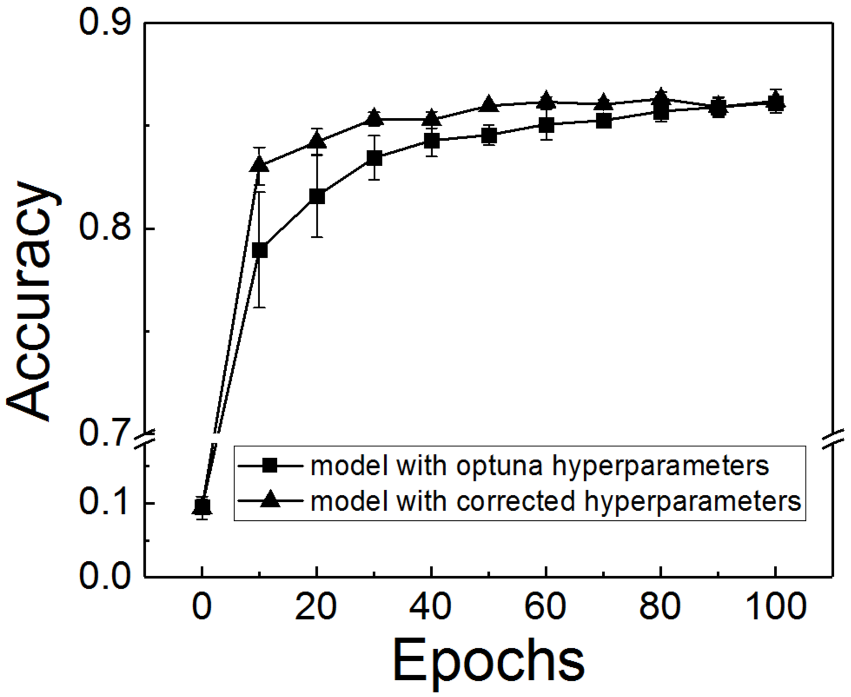

Figure S3. Software modeling of the CNN with binarized F-MNIST and trainable filter for different CNN hyperparameters (Optuna and manually fine-tuned).

Table S1. The Optuna-optimized hyperparameters and manually fine-tuned hyperparameters.

Parameter name	Optuna hyperparameters	Corrected

		**hyperparameters**
Image binarization threshold	10 (estimated by eye)	
Batch size	256 (estimated based on the time and memory consumption)	
Number of fully connected layers	2	
Neurons in hidden layer	64	32
Weights initialization	Normal distribution (mean = 0, std = 0.1)	
Activation function	Sigmoid	
Loss function	CrossEntropyLoss	
Learning rate	0.17	1

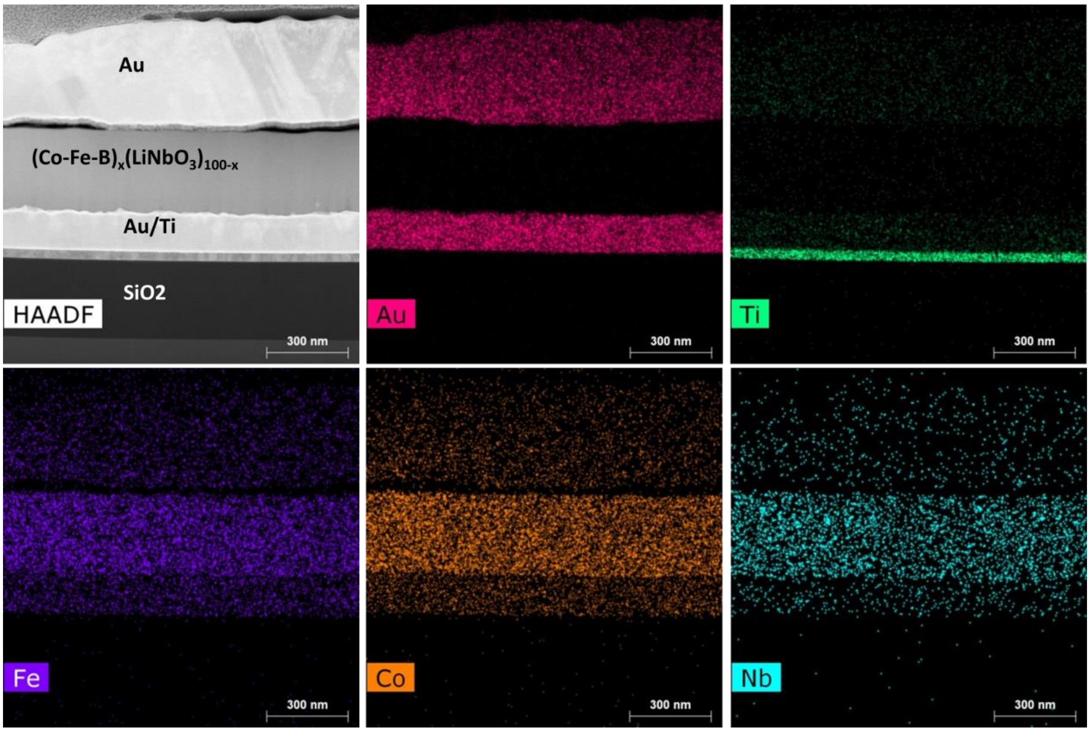

Figure S4. Elemental EDX-maps of the M/NC/LNO/M memristor from a crossbar array.

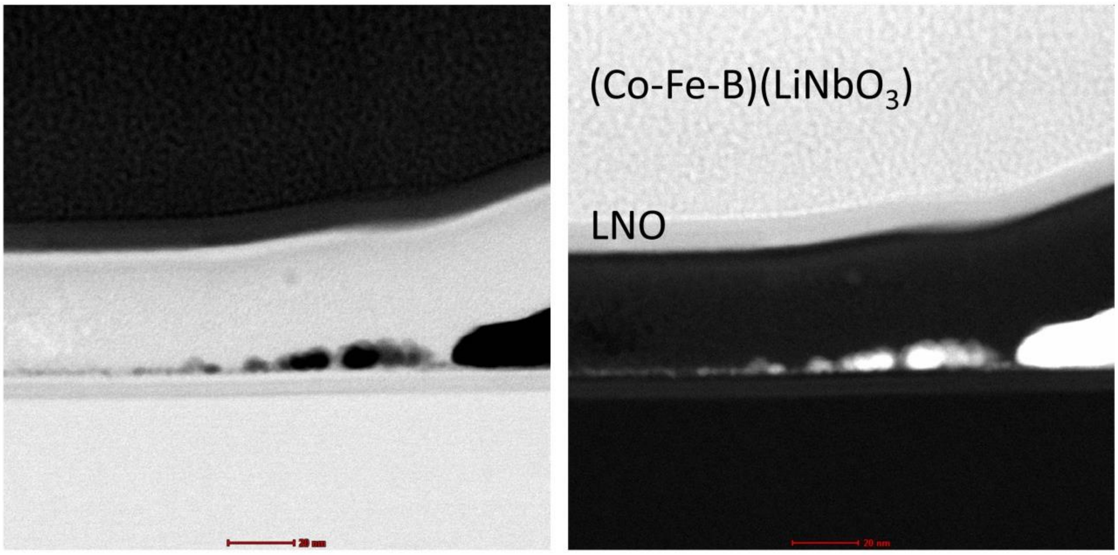

Figure S5. High resolution dark field TEM image of the area near the bottom electrode (at the edge of the crossbar busses intersection) of the M/NC/LNO/M memristor from a crossbar array.

Table S2. The influence of the introduced variation on the training process of the 2-kernel hybrid CNN.

Coefficient of variation (%)	Accuracy score for the test dataset (%)		
	Mean	Min	Max
0	84	82	85
1	83	80	85
10	82	78	84
20	81	79	83
…			
100	71	69	73

References:

[39] T. Akiba, S. Sano, T. Yanase, T. Ohta, M. Koyama, *KDD '19 Proc. 25th ACM SIGKDD Int. Conf. Knowl. Discov. Data Min.* **2019**, 2623.
